# Burkholderia Type VI Secretion Systems Have Distinct Roles in Eukaryotic and Bacterial Cell Interactions

**DOI:** 10.1371/journal.ppat.1001068

**Published:** 2010-08-26

**Authors:** Sandra Schwarz, T. Eoin West, Frédéric Boyer, Wen-Chi Chiang, Mike A. Carl, Rachel D. Hood, Laurence Rohmer, Tim Tolker-Nielsen, Shawn J. Skerrett, Joseph D. Mougous

**Affiliations:** 1 Department of Microbiology, University of Washington, Seattle, Washington, United States of America; 2 Department of Medicine, University of Washington, Seattle, Washington, United States of America; 3 UMR754 INRA-ENVL-UCBL-EPHE “Rétrovirus et Pathologie Comparée”, IFR 128 BioSciences Lyon-Gerland, Université Claude Bernard Lyon 1, Lyon, France; 4 Department of International Health, Immunology and Microbiology, University of Copenhagen, Copenhagen, Denmark; 5 Department of Immunology, University of Washington, Seattle, Washington, United States of America; University of Texas-Houston Medical School, United States of America

## Abstract

Bacteria that live in the environment have evolved pathways specialized to defend against eukaryotic organisms or other bacteria. In this manuscript, we systematically examined the role of the five type VI secretion systems (T6SSs) of *Burkholderia thailandensis* (*B. thai*) in eukaryotic and bacterial cell interactions. Consistent with phylogenetic analyses comparing the distribution of the *B. thai* T6SSs with well-characterized bacterial and eukaryotic cell-targeting T6SSs, we found that T6SS-5 plays a critical role in the virulence of the organism in a murine melioidosis model, while a strain lacking the other four T6SSs remained as virulent as the wild-type. The function of T6SS-5 appeared to be specialized to the host and not related to an *in vivo* growth defect, as ΔT6SS-5 was fully virulent in mice lacking MyD88. Next we probed the role of the five systems in interbacterial interactions. From a group of 31 diverse bacteria, we identified several organisms that competed less effectively against wild-type *B. thai* than a strain lacking T6SS-1 function. Inactivation of T6SS-1 renders *B. thai* greatly more susceptible to cell contact-induced stasis by *Pseudomonas putida*, *Pseudomonas fluorescens* and *Serratia proteamaculans*—leaving it 100- to 1000-fold less fit than the wild-type in competition experiments with these organisms. Flow cell biofilm assays showed that T6S-dependent interbacterial interactions are likely relevant in the environment. *B. thai* cells lacking T6SS-1 were rapidly displaced in mixed biofilms with *P. putida*, whereas wild-type cells persisted and overran the competitor. Our data show that T6SSs within a single organism can have distinct functions in eukaryotic versus bacterial cell interactions. These systems are likely to be a decisive factor in the survival of bacterial cells of one species in intimate association with those of another, such as in polymicrobial communities present both in the environment and in many infections.

## Author Summary

Many bacteria encounter both eukaryotic cells and other bacterial species as a part of their lifestyles. In order to compete and survive, these bacteria have evolved specialized pathways that target these distinct cell types. Type VI secretion systems (T6SSs) are bacterial protein export machines postulated to puncture targeted cells using an apparatus that shares structural similarity to bacteriophage. We investigated the role of the five T6SSs of *Burkholderia thailandensis* in the defense of the organism against other bacteria and higher organisms. *B*. *thailandensis* is a relatively avirulent soil saprophyte that is closely related to the human pathogen *B. pseudomallei*. Our work uncovered roles for two *B. thailandensis* T6SSs with specialized functions either in the survival of the organism in a murine host, or against another bacterial cell. We also found that *B. thailandensis* lacking the bacterial-targeting T6SS could not persist in a mixed biofilm with a competing bacterium. Based on the evolutionary relationship of T6SSs, and our findings that *B. thailandensis* engages other bacterial species in a T6S-dependent manner, we speculate that this pathway is of general significance to interbacterial interactions in polymicrobial human diseases and the environment.

## Introduction

Bacteria have evolved many mechanisms of defense against competitors and predators in their environment. Some of these, such as type III secretion systems (T3SSs) and bacteriocins, provide specialized protection against eukaryotic or bacterial cells, respectively [1], [2]. Gene clusters encoding apparent type VI secretion systems (T6SSs) are widely dispersed in the proteobacteria; however, the general roles of these systems in eukaryotic versus bacterial cell interactions are not known [3], [4].

To date, most studies of T6S have focused on its role in pathogenesis and host interactions [5], [6], [7]. In certain instances, compelling evidence for the specialization of T6S in guiding eukaryotic cell interactions has been generated. Most notably, the systems of *Vibrio cholerae* and *Aeromonas hydrophila* were shown to translocate proteins with host effector domains into eukaryotic cells [8], [9]. Evidence is also emerging that T6SSs could contribute to interactions between bacteria. The *Pseudomonas aeruginosa* HSI-I-encoded T6SS (H1-T6SS) was shown to target a toxin to other *P. aeruginosa* cells, but not to eukaryotic cells [10]. Unfortunately, analyses of the ecological niche occupied by bacteria that possess T6S have not been widely informative for classifying their function [3], [4]. These efforts are complicated by the fact that pathogenic proteobacteria have environmental reservoirs, where they undoubtedly encounter other bacteria. The observation that many bacteria possess multiple evolutionarily distinct T6S gene clusters–up to six in one organism–raises the intriguing possibility that each system may function in an organismal or context-specific manner [3].

The T6SS is encoded by approximately 15 core genes and a variable number of non-conserved accessory elements [4]. Data from functional assays and protein localization studies suggest that these proteins assemble into a multi-component secretory apparatus [11], [12], [13]. The AAA+ family ATPase, ClpV, is one of only a few core proteins of the T6S apparatus that have been characterized. Its ATPase activity is essential for T6S function [14], and it associates with several other conserved T6S proteins [15], [16]. ClpV-interacting proteins A and B (VipA and VipB) form tubules that are remodeled by the ATPase, which could indicate a role for the protein in secretion system biogenesis. Two proteins exported by the T6SS are haemolysin co-regulated protein (Hcp) and valine-glycine repeat protein G (VgrG). Secretion of these proteins is co-dependent, and they may be extracellular components of the apparatus [10], [13], [17], [18], [19], [20].

*Burkholderia pseudomallei* is an environmental saprophyte and the causative agent of melioidosis [21]. Infection with *B. pseudomallei* typically occurs percutaneously via direct contact with contaminated water or soil, however it can also occur through inhalation. The ecological niche and geographical distribution of *B. pseudomallei* overlap with a relatively non-pathogenic, but closely related species, *Burkholderia thailandensis* (*B. thai*) [22]. The genomes of these bacteria are highly similar in both overall sequence and gene synteny [23], [24]. One study estimates that the two microorganisms separated from a common ancestor approximately 47 million years ago [24]. It is postulated that the *B. pseudomallei* branch then diverged from *Burkholderia mallei*, which underwent rapid gene loss and decay during its evolution into an obligate zoonotic pathogen [25]. As closely related organisms that represent three extremes of bacterial adaptation, this Burkholderia group offers unique insight into the outcomes of different selective pressures on the expression and maintenance of certain traits.

*B. pseudomallei* possesses a large and complex repertoire of specialized protein secretion systems, including three T3SSs and six evolutionarily distinct T6SSs [3], [26], [27]. The genomes of *B. thailandensis* and *B. mallei* contain unique sets of five of the six *B. pseudomallei* T6S gene clusters; thus, of the six evolutionarily distinct “Burkholderia T6SSs,” four are conserved among the three species. Remarkably, T6SSs account for over 2% of the coding capacity of the large genomes of these organisms. For the current study, we have adopted the Burkholderia T6SS nomenclature proposed by Shalom and colleagues [28].

To date, only Burkholderia T6SS-5, one of the four conserved systems, has been investigated experimentally. The system was investigated in *B. mallei* based on its co-regulation with virulence determinants such as actin-based motility and capsule [27]. *B. mallei* strains lacking a functional T6SS-5 are strongly attenuated in a hamster model of glanders. Preliminary studies suggest that T6SS-5 is also required for *B. pseudomallei* pathogenesis [28], [29]. In one study, a strain bearing a transposon insertion within T6SS-5 was identified in a screen for *B. pseudomallei* mutants with impaired intercellular spreading in cultured epithelial cells [29]. The authors also showed that this insertion caused significant attenuation in a murine infection model.

Herein, we set out to systematically define the function of the Burkholderia T6SSs. Our study began with the observation that well-characterized examples of eukaryotic and bacterial cell-targeting T6SSs segregate into distant subtrees of the T6S phylogeny. We found that Burkholderia T6SS-5 clustered closely with eukaryotic cell-targeting systems, and was the only system in *B. thai* that was required for virulence in a murine model of pneumonic melioidosis. The remaining systems clustered proximally to a bacterial cell-targeting T6SS in the phylogeny. One of these, T6SS-1, displayed a profound effect on the fitness of *B. thai* in competition with several bacterial species. The function of T6SS-1 required cell contact and its absence caused sensitivity of the strain to stasis induced by competing bacteria. In flow cell biofilm assays initiated with 1∶1 mixtures of *B. thai* and *Pseudomonas putida*, wild-type *B. thai* predominated, whereas the ΔT6SS-1 strain was rapidly displaced by *P. putida*. Our findings point toward an important role for T6S in interspecies bacterial interactions.

## Results

### Phylogenetic analysis of T6SSs

**Figure 1:**
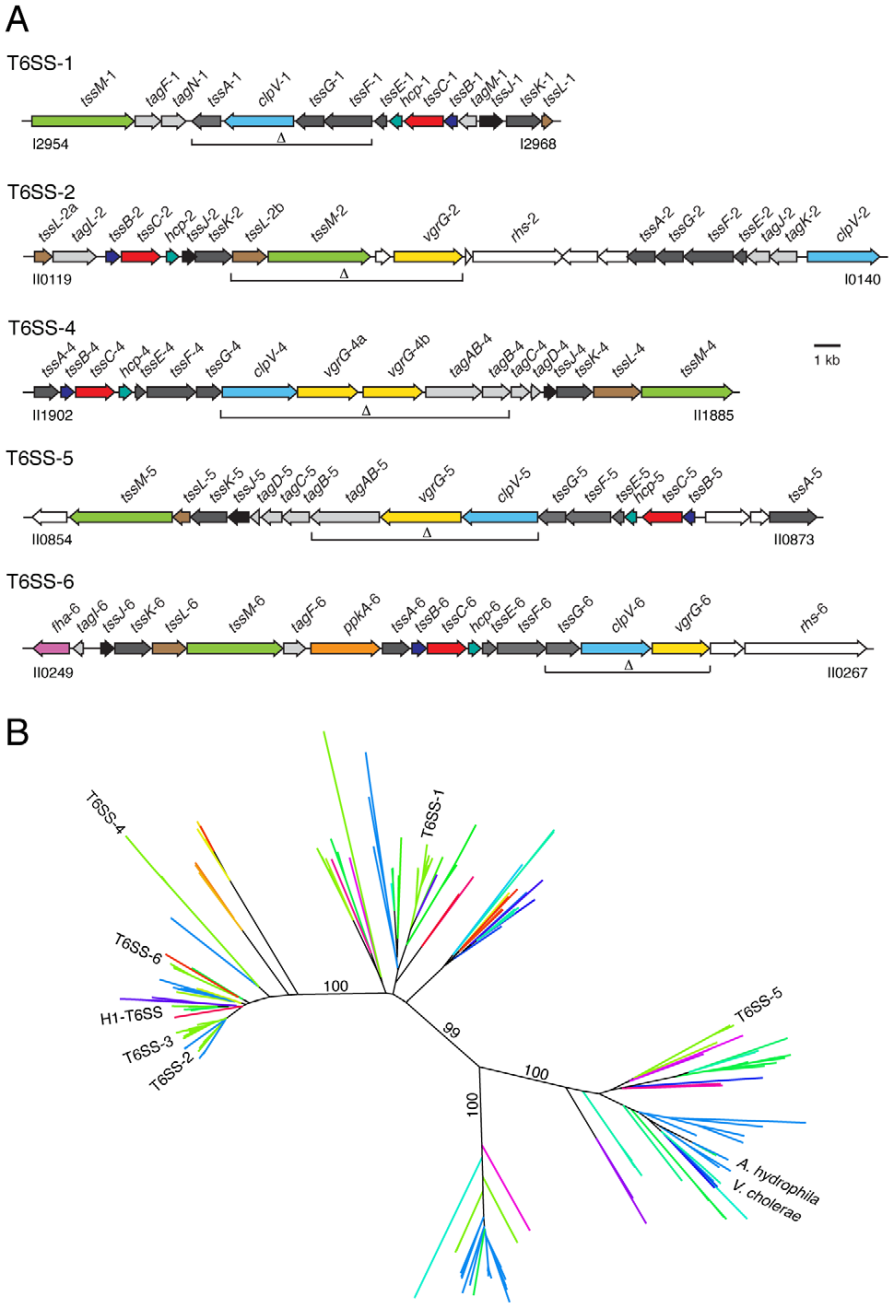
The Burkholderia T6SSs cluster with eukaryotic and prokaryotic-targeting systems in a T6S phylogeny. (A) Overview of the *B. thai* T6SS gene clusters. Burkholderia T6SS-3 is absent from *B.thai*. Genes were identified according to the nomenclature proposed by Shalom and colleagues [28]: *tss*, type six secretion conserved genes; *tag*, type six secretion-associated genes variably present in T6SSs. Genes are colored according to function and conservation (dark grey, *tss* genes; light grey, *tag* genes; color, experimentally characterized *tss* or *tag* genes; white, genes so far not linked to T6S). Brackets demarcate genes that were deleted in order to generate *B. thai* strains ΔT6SS-1, -2, -4 -5 and -6 and their assorted combinations. Locus tag numbers are based on *B. thai* E264 genome annotations. (B) Neighbor-joining tree based on 334 T6S-associated VipA orthologs. The locations of VipA proteins from T6SSs discussed in the text are indicated. Each line represents one or more orthologous T6SSs from a single species. Lines are colored based on bacterial taxonomy of the corresponding organism. Indicated bootstrap values correspond to 100 replicates. This phylogeny is available in expanded format in Figure S1. A key for the coloring scheme is also present in Figure S1.

We conducted phylogenetic analyses of all available T6SSs to examine the evolutionary relationship between eukaryotic and bacterial cell-targeting systems. The phylogenetic tree we constructed was based on VipA, as this protein is a highly conserved element of T6SSs that has been demonstrated to physically interact with two other core T6S proteins, including the ClpV ATPase [15]. In the resulting phylogeny, the systems of *V. cholerae* and *A. hydrophila*, two well-characterized eukaryotic cell-targeting systems, clustered closely within one of the subtrees, whereas the bacteria-specific *P. aeruginosa* H1-T6SS was a member of a distant subtree (Figure 1 and see Figure S1) [8], [9], [10]. In an independent analysis, Bingle and colleagues observed a similar T6S phylogeny, and termed these subtrees “D” and “A,” respectively [3].

Next we examined the locations of the six Burkholderia T6SSs. Interestingly, T6SS-5, the only Burkholderia system previously implicated in virulence, clustered within the substree containing the *V. cholerae* and *A. hydrophila* systems (Figure 1). Four of the remaining Burkholderia systems clustered within the subtree that included the H1-T6SS, and the final system was found in a neighboring subtree. These data led us to hypothesize that T6SSs of differing organismal specificities are evolutionarily distinct. Apparent contradictions between organismal specificity based on our phylogenetic distribution and studies demonstrating T6S-dependent phenotypes were identified, however these instances are difficult to interpret because specificity was not measured and cannot be ascertained from available data.

### T6SS-5 is required for virulence; systems 1, 2, 4 and 6 are dispensible

We chose *B. thai* as a tractable model organism in which to experimentally investigate the role of the Burkholderia T6SSs. Due to our limited knowledge regarding the function and essentiality of each gene within a given T6SS cluster, we reasoned it prudent to inactivate multiple conserved genes for initial phenotypic studies. Strains lacking the function of each of the five *B. thai* T6SSs (Burkholderia T6SS-3 is absent in *B. thai*) were prepared by removing three to five genes, including at least two that are highly conserved (Figure 1A). When possible, polar effects were minimized by deleting from a central location in each cluster.

**Figure 2:**
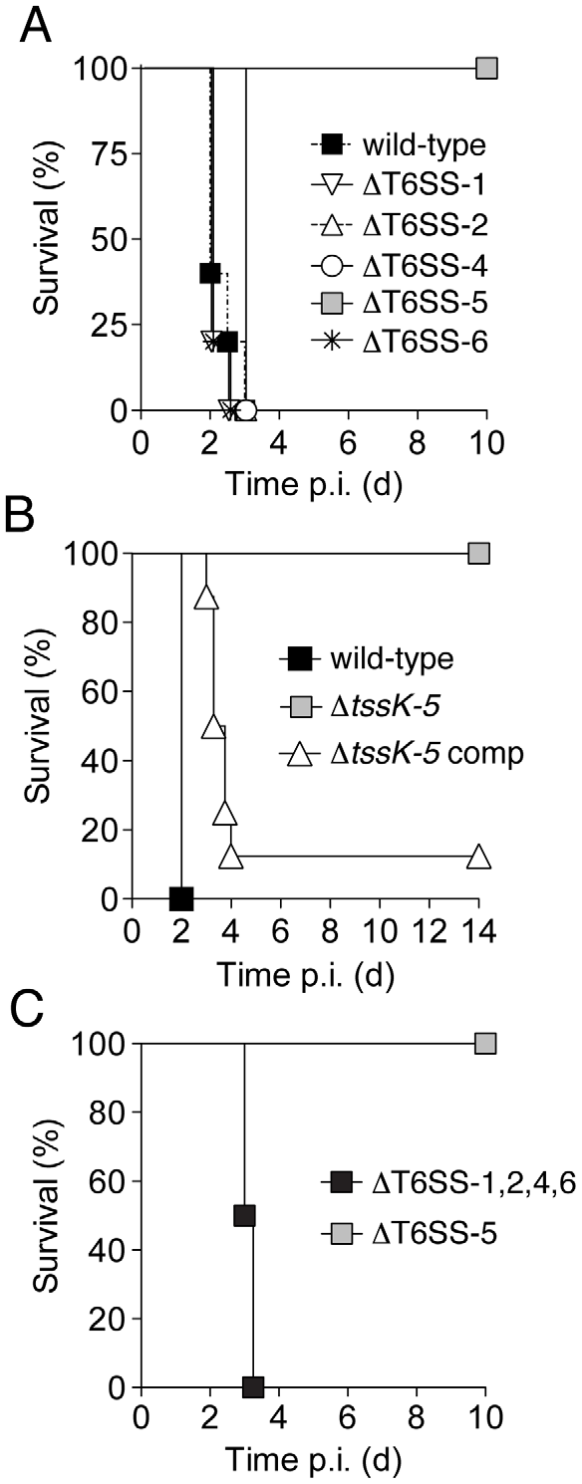
Of the five *B. thai* T6SSs, only T6SS-5 is required for virulence in a murine acute melioidosis model. C57BL/6 wild-type mice were infected by the aerosol-route with 10^5^ c.f.u./lung of *B. thai* strains and monitored for survival for 10–14 days post infection (p.i.). Survival of mice after exposure to *B. thai* (A) wild-type and strains harboring gene deletions in individual T6SS gene clusters (n = 5 per group), (B) wild-type and a strain bearing an in-frame *tssK-5* deletion (Δ*tssK-5*) or its complemented derivative (Δ*tssK-5*-comp; n = 7, 7 and 8, respectively), (C) or a strain with inactivating mutations in T6SS-5 or in four T6SSs (ΔT6SS-1,2,4,6; n = 6 and 8, respectively).

To probe the role of the Burkholderia T6SSs in virulence, we utilized a recently developed acute pneumonia model of melioidosis [30]. The survival of mice infected with approximately 10^5^ aerosolized wild-type or mutant bacteria was monitored over the course of ten days. Consistent with previous studies implicating T6SS-5 in *B. mallei* and *B. pseudomallei* pathogenesis, mice infected with ΔT6SS-5 survived the course and displayed no outward symptoms of the infection (Figure 2A) [27], [29]. On the other hand, those infected with the wild-type strain or strains bearing deletions in the other T6SSs succumbed by three days post infection (p.i.).

The *B. thai* T6SS-5 locus is adjacent to *bsa* genes, which encode an animal pathogen-like T3SS. Inactivation of the *bsa* T3SS secretion system also leads to dramatic attenuation of *B. thai* in the model we utilized [26]. The regulation of these secretion systems appears to be intertwined; a recent study in *B. pseudomallei* showed that a protein encoded within the *bsa* cluster strongly activates T6SS-5 of that organism [31]. To rule out the possibility that attenuation of ΔT6SS-5 was attributable to polar effects or changes in regulation of the *bsa* T3SS, we generated a strain bearing an in-frame deletion of a single gene in the cluster, *tssK-5* (Figure 1A). A *tssK-5* ortholog is readily identified in nearly all T6S gene clusters and it shares no homology with known regulators. Like the T6SS-5 deletion, Δ*tssK-5* completely attenuated the organism (Figure 2B). Genetic complementation of this phenotype further confirmed that T6SS-5 is an essential virulence factor of the organism.

To investigate whether the retention of virulence in the ΔT6SS-1,2,4 and 6 strains could be attributed to either compensatory activity or redundancy, we next constructed a strain bearing inactivating mutations in all four clusters and measured its virulence in mice. Mice infected with this strain succumbed to the infection with similar kinetics to those infected with the wild-type, indicating that T6SS-5 is the only system of *B. thai* that is required for virulence in this model (Figure 2C). In summary, these data indicate that T6SS-5 is a major virulence factor for *B. thai* in a murine acute melioidosis model, whereas the remaining putative T6SSs of the organism are dispensible for virulence.

### Burkholderia T6SS-5 plays a specific role in host interactions

**Figure 3:**
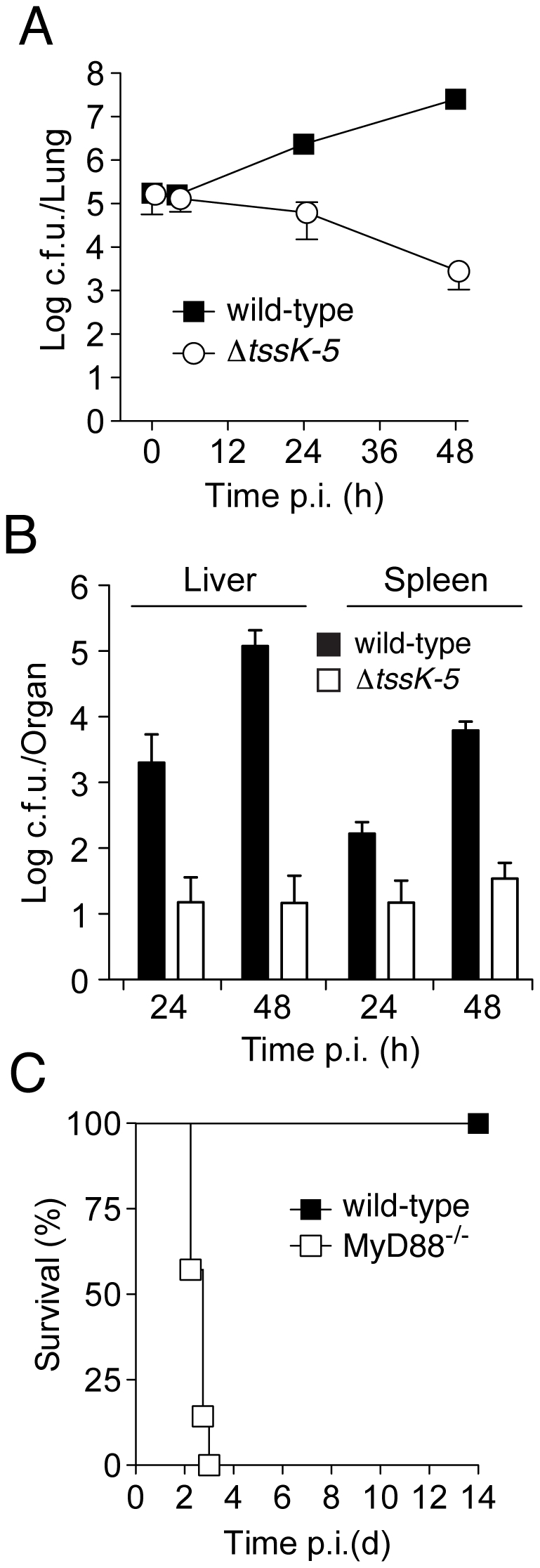
*B. thai* Δ*tssK-5* shows a replication defect in the lung of wild-type mice but is highly virulent in MyD88^−/−^ mice. Mice were exposed to 10^5^ c.f.u./lung aerosolized *B. thai* wild-type or Δ*tssK-5* bacteria and c.f.u. were monitored in the (A) lung after 4, 24, and 48 h (n = 6 per time point), and in the (B) liver and spleen after 24 and 48 h (n = 6 per time point). (C) C57BL/6 wild-type (n = 6) and MyD88^−/−^ mice (n = 7) were infected with the Δ*tssK-5* strain and survival was monitored for 14 days. Error bars in (A) and (B) are ± SD.

To more closely examine the requirement for T6SS-5 during infection, we monitored *B. thai* wild-type and Δ*tssK-5* c.f.u. in the lung, liver, and spleen at 4, 24, and 48 hours following inoculation with approximately 10^5^ bacteria by aerosol. At 4 hours p.i., no differences were observed in c.f.u. recovered from the lung (Figure 3A). After this initial phase, lung c.f.u. of Δ*tssK-*5 gradually declined, whereas wild-type populations expanded approximately 100-fold. Both organisms spread systemically, however significantly fewer Δ*tssK-5* cells were recovered from the liver and spleen at 24 and 48 hours p.i. (Figure 3B).

Thus far, our findings did not distinguish between a specific role for T6SS-5 in host interactions, such as escaping or manipulating the innate immune system, versus the alternative explanation that T6SS-5 is generally required for growth in host tissue. To discriminate between these possibilities, we compared the virulence of Δ*tssK-5* in wild-type mice to a strain with compromised innate immunity, MyD88^−/−^ [32], [33]. Mice lacking MyD88 were unable to control the Δ*tssK-5* infection and succumbed within 3 days (Figure 3C). The differences in virulence of the Δ*tssk-5* strain in wild-type and MyD88^−/−^ infections suggest that T6SS-5 is required for effective defense of the bacterium against one or more innate immune responses of the host. Altogether, these data strongly support the conclusion that T6SS-5 has evolved to play a specific role in the fitness of *B. thai* in a eukaryotic host environment.

### T6S impacts the fitness of *B. thai* in co-culture with diverse bacterial species

Earlier work by our laboratory has shown that T6S can influence intraspecies bacterial interactions. We showed that the H1-T6SS of *P. aeruginosa* targets a toxin to other *P. aeruginosa* cells [10], and that in growth competition assays, toxin-secreting strains are provided a fitness advantage relative to strains lacking a specific toxin immunity protein. Based on this information and the locations of the *B. thai* T6SSs within our phylogeny, we postulated that one or more of these systems could also play a role in interbacterial interactions. Preliminary studies indicated that T6S did not influence interactions between *B. thai* strains, thus we decided to test the hypothesis that the *B. thai* T6SSs play a role in interspecies bacterial interactions.

**Figure 4:**
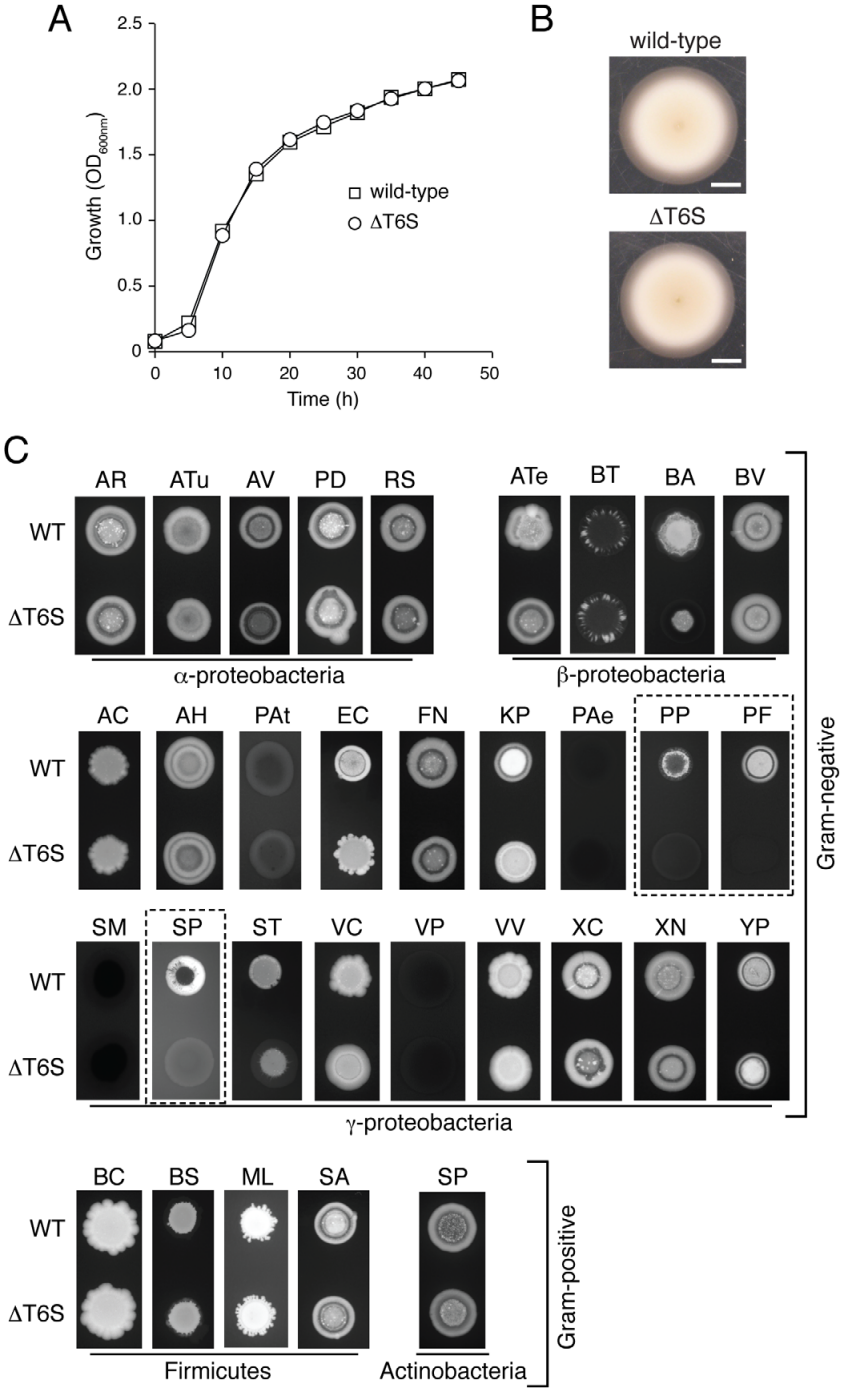
T6S plays a role in the fitness of *B. thai* in growth competition assays with other bacteria. (A) *In vitro* growth of *B. thai* wild-type and a strain bearing gene deletions in all five T6SSs (ΔT6S). The data presented are an average of three replicates. (error bars smaller than symbols). (B) *B. thai* wild-type and ΔT6S swimming motility in semi-solid LB agar (scale bar = 1.0 cm). (C) Fluorescence images of growth competition assays between GFP-labeled *B. thai* wild-type and ΔT6S strains against the indicated unlabeled competitor species. Competition assay outcomes could be divided into T6S-independent (AR, *Agrobacterium rhizogenes*; ATu, *A. tumefaciens*; AV, *A. vitis*; PD, *Paracoccus denitrificans*; RS, *Rhodobacter sphaeroides*; ATe, *Acidovorax temperans*; BT, *B. thailandensis*; BV, *B. vietnamiensis*; AC, *Acinetobacter calcoaceticus*; AH, *Aeromonas hydrophila*; PAt, *Pectobacterium atrosepticum*; FN, *Francisella novicida*; PAe, *Pseudomonas aeruginosa*; SM, *Serratia marcescens*; VC, *Vibrio cholerae*; VP, *Vibrio parahaemolyticus*; VV, *V. vulnificus*; XC, *Xanthomonas campestris*; XN, *Xenorhabdus nematophilus*; YP, *Yersinia pestis* LCR^–^; BC, *Bacillus cereus*; BS, *B. subtilis*; ML, *Micrococcus luteus*; SA, *Staphylococcus aureus*; SP, *Streptococcus pyogenes*), those with modest T6S-effects (BA, *B. ambifaria*; EC, *E. coli*; KP, *Klebsiella pneumoniae*; ST, *Salmonella typhimurium*) and those in which *B. thai* proliferation was strongly T6S-dependent (dashed boxes – PP, *P. putida* E0044; PF, *P. fluorescens* ATCC27663; SP, *S* ***.*** *proteamaculans* 568). This latter group of organisms is referred to as the T6S-dependent competitors (TDCs).

Without information to guide predictions of specificity, we developed a simple and relatively high-throughput semi-quantitative assay to allow screening of a wide range of organisms for sensitivity to the *B. thai* T6SSs. The design of the assay was based on two key assumptions for T6S-dependent effects – that they are cell contact-dependent and that they impact fitness (as measured by proliferation). To facilitate measurement of T6S-dependent changes in *B. thai* proliferation in the presence of competing organisms, we engineered constitutive green fluorescent protein expression cassettes into wild-type *B. thai* and a strain bearing mutations in all five T6SSs (ΔT6S) [34]. Control experiments showed that the lack of T6S function did not impact growth or swimming motility (Figure 4A and 4B). To test the assay, we conducted competition experiments between the GFP-labeled wild-type and ΔT6S strains against the unlabeled wild-type strain. The GFP-expressing cells were clearly visualized in the mixtures, and, importantly, wild-type and ΔT6S competed equally with the parental strain (Figure 4C; BT).

We next screened the *B. thai* strains against 31 species of bacteria. Most of these were Gram-negative proteobacteria (5α; 3β; 18γ), however two Gram-positive phyla were also represented (4 Firmicutes; 1 Actinobacteria). Although we endeavored to screen a large diversity of bacteria, many taxa could not be included due to specific nutrient requirements or an unacceptably slow growth rate under the conditions of the assay (30°C, Luria-Bertani (LB) medium). The outcomes of most competition experiments were independent of the T6SSs of *B. thai*. T6S-independent outcomes varied; in most instances, *B. thai* flourished in the presence of the competing organism (Figure 4C). However, a small subset of species markedly inhibited *B. thai* growth (Figure 4C; PAt, PAe, SM, VP). Interestingly, *B. thai* proliferation was reproducibly affected in a T6S-dependent manner in competition experiments against 7 of the 31 species tested. All of these were Gram-negative organisms, and in each case, *B. thai* ΔT6S was less fit than the wild-type. T6S-dependent competition outcomes fell into two readily discernable groups; the first included three γ- and one β-proteobacteria (Figure 4C; BA, EC, KP, ST). In competition with these organisms, *B. thai* ΔT6S displayed only a modest decrease in proliferation relative to the wild-type. Differences in the size and morphology of assay “spots” containing wild-type or ΔT6S were noted in several instances for this group of organisms. Quantification of c.f.u. verified that these differences were reflective of a minor, but highly reproducible fitness defect of ΔT6S (data not shown).

The second group consisted of three γ-proteobacteria: *P. putida*, *P. fluorescens*, and *S. proteamaculans*. The proliferation of *B. thai* grown in competition with these organisms appeared to be highly dependent on T6S (Figure 4C; PP, PF, SP). For further analyses, we focused on this latter group; henceforth referred to as the “T6S-dependent competitors” (TDCs).

### T6SS-1 is involved in cell contact-dependent interbacterial interactions

**Figure 5:**
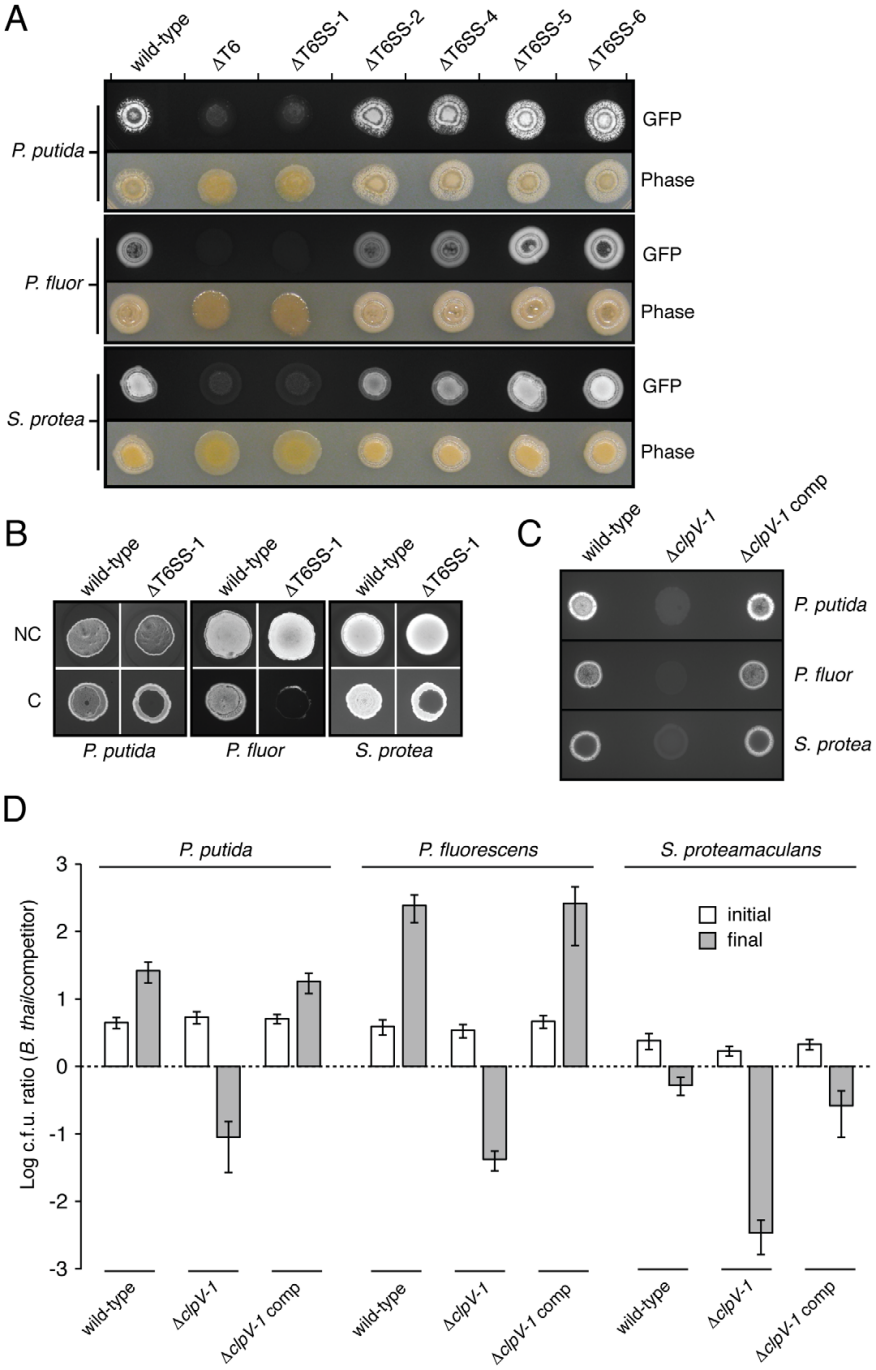
T6SS-1 is involved in cell contact-dependent interbacterial interactions. (A) Growth competition assays between the indicated GFP-labeled *B. thai* strains and the TDCs. Standard light photographs and fluorescent images of the competition assays are shown. (B) Fluorescence images of GFP-labeled *B. thai* wild-type and ΔT6SS-1 grown in the presence of the TDCs with (no contact, NC) or without (contact, C) an intervening filter. (C) Fluorescence images of growth competition assays between GFP-labeled *B. thai* Δ*clpV-1* or complemented Δ*clpV-1* with the TDCs. (D) Quantification of c.f.u before (initial) and after (final) growth competition assays between the indicated organisms. The c.f.u. ratio of the *B. thai* strain versus competitor bacteria is plotted. Error bars represent ± SD.

The next question we addressed was whether one or more of the individual T6SSs were responsible for the TDC-specific proliferation phenotype of *B. thai* ΔT6S. To determine this, we inserted a GFP over-expression cassette into our panel of individual *B. thai* T6SS deletion strains, and performed plate competition assays against the TDCs. In competition with each TDC, ΔT6SS-1 appeared as deficient in proliferation as ΔT6S, whereas the other strains grew similarly to the wild-type (Figure 5A). The dramatic differences in the competition outcomes between the strains were also discernable by the naked eye. Competition experiments that included *B. thai* lacking T6SS-1 had a morphology similar to a mono-culture of the TDC, whereas co-cultures possessing an intact T6SS-1 were more similar in appearance to *B. thai* mono-culture.

It remained possible that the effects of T6SS-1 on the fitness of *B. thai* in competition with other bacteria were either non-specific or unrelated to its putative role as a T6SS. As mentioned earlier, one common observation from detailed studies of T6SSs conducted to date is that its effects require cell contact [8], [9], [10]. This has been postulated to reflect a conserved mechanism of the apparatus akin to bacteriophage cell puncturing [18]. To address whether the apparent fitness defect of ΔT6SS-1 involves a mechanism consistent with T6S, we probed whether its effects were dependent upon cell contact. A filter (0.2 µm pore diameter) placed between *B. thai* and TDC cells abrogated the T6SS-1-dependent growth defect (Figure 5B). In control experiments, the three TDCs were directly applied to an underlying layer of the *B. thai* strains. In each case, a zone of clearing was observed in the ΔT6SS-1 layer, while no effect on wild-type proliferation was noted. From these data we conclude that cell contact is essential for the activity of T6SS-1.

We next sought to quantify the magnitude of T6SS-1 effects on *B. thai* fitness in competition with TDCs. To ensure the specificity of T6SS-1 inactivation in the strains used in these assays, we generated a *B. thai* strain bearing an in-frame *clpV-1* deletion, and a strain in which this deletion was complemented by *clpV-1* expression from a neutral site on the chromosome. In plate competition assays, the Δ*clpV-1* strain displayed a fitness defect similar to ΔT6SS-1, and *clpV-1* expression complemented the phenotype (Figure 5C). Measurements comparing *B. thai* and TDC c.f.u. in the competition assay inoculum to material recovered from the assays following several days of incubation confirmed that inactivation of T6SS-1 leads to a dramatic fitness defect of *B. thai* (Figure 5D). Depending on the TDC, the competitive index (c.i.; final c.f.u. ratio/initial c.f.u ratio) of wild-type *B. thai* was approximately 120-5,000-fold greater than that of the Δ*clpV-1* strain. All TDCs out-competed Δ*clpV-1* (0.0021<c.i. <0.015); on the contrary, wild-type *B. thai* was highly competitive against *P. putida* (c.i.: 5.8) and *P. fluorescens* (c.i.: 61), and its relative numbers decreased only modestly in assays with *S. proteamaculans* (c.i.: 0.24). In summary, our findings indicate that T6SS-1 plays an important role in the interactions of *B. thai* cells in direct contact with other bacteria. T6SS-1-dependent effects are species-specific, and in some cases, can be a major determinant of *B. thai* proliferation.

### T6SS-1 provides resistance to *P. putida* induced stasis of *B. thai*

**Figure 6:**
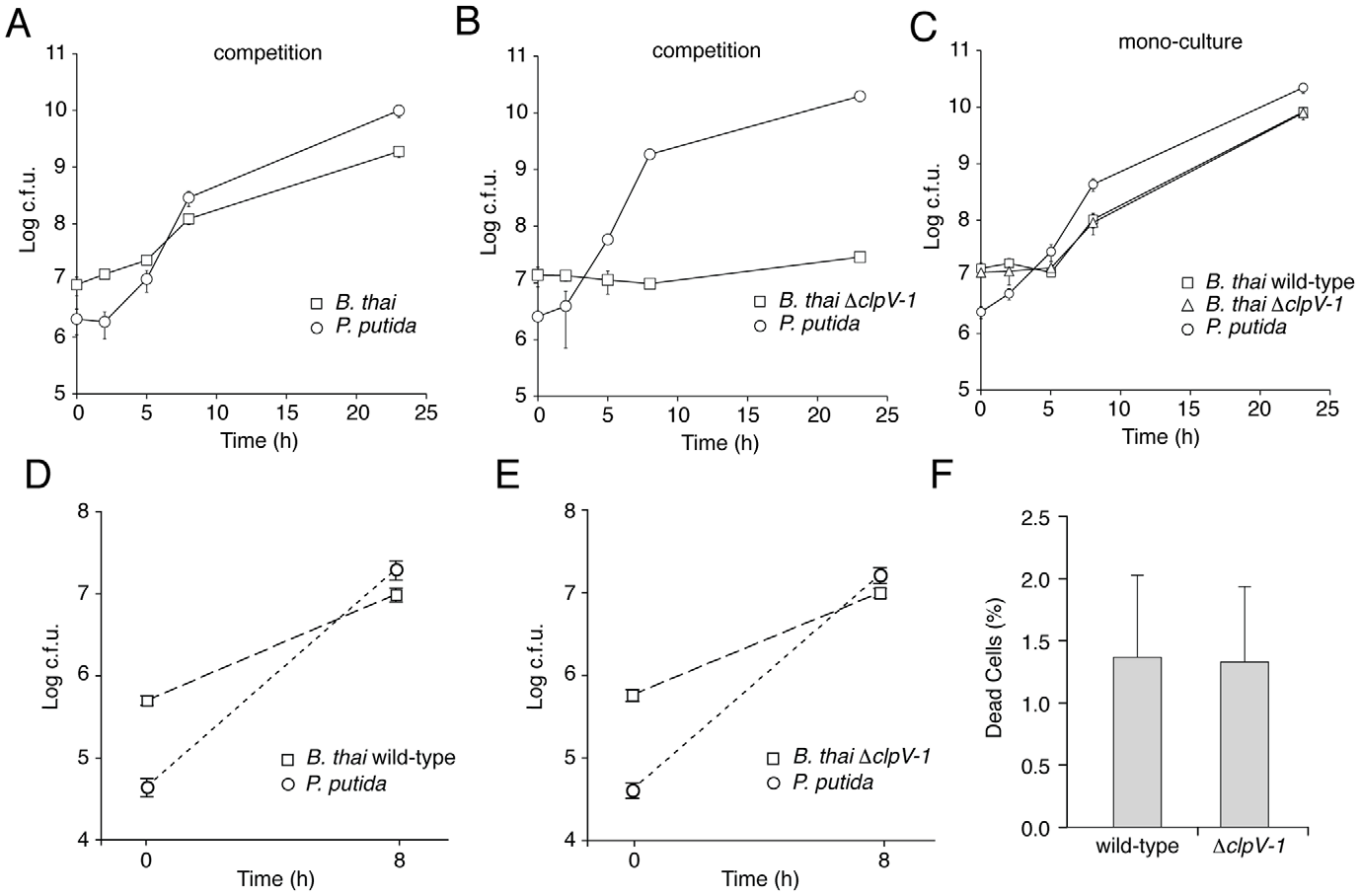
T6SS-1 is required for resistance against *P. putida*-induced growth inhibition. (A–C) *B. thai* and *P. putida* growth following inoculation of competitive cultures (A, B) or mono-cultures (C) onto LB 3% w/v agar. (D, E) *B. thai* and *P. putida* growth following inoculation of competitive cultures into LB broth. (F) Quantification of dead cells 7.5 hours after initiating competition between *P. putida* and the indicated *B. thai* strain on LB 3% w/v agar (n≥7,000). Error bars are ± SD.

Three models could explain the T6SS-1-dependent effects we observed on *B. thai* fitness in competition with the TDCs: (i) T6SS-1 inhibits TDC proliferation, thereby freeing nutrients for *B. thai*; (ii) T6SS-1 prevents TDC inhibition of *B. thai* growth; or (iii) T6SS-1 performs both of these functions. To distinguish between these possibilities, we compared *B. thai* and TDC growth rates following inoculation into either mono-culture or competitive cultures on 3% agar plates. Our prior experiments indicated that T6SS-1-dependent effects on *B. thai* were similar in competition assays with each TDC (Figure 4F and Figure 5), therefore we utilized *P. putida* to represent the TDCs in this and subsequent experiments. Surprisingly, we found that the proliferation of *P. putida* and wild-type *B. thai* was largely unaffected in competition assays (Figure 6A–C). However, Δ*clpV-1* proliferation was severely hampered in the presence of *P. putida*. Indeed, *B. thai* Δ*clpV-1* c.f.u. expanded by only 2.1-fold during the first 23 hours of the experiment, whereas wild-type c.f.u. increased 220-fold. Consistent with earlier results in *P. aeruginosa* [10], the effects of T6SS-1 on the fitness of *B. thai* in co-culture with *P. putida* were not observed in liquid medium (Figure 6D and 6E).

The proliferation defect of *B. thai* Δ*clpV-1* could be attributable to *P. putida*-induced growth inhibition, cell killing, or a combination of these factors. We reasoned that if killing was involved in the Δ*clpV-1* phenotype, the difference in cell death between wild-type and Δ*clpV-1* would be most pronounced at approximately 7.5 hours following inoculation of the competition assays, when wild-type *B. thai* are rapidly proliferating and Δ*clpV-1* cell numbers are not expanding. At this time point, we identified similar numbers of dead cells in wild-type and Δ*clpV-1* competitions, suggesting that T6SS-1 inhibits stasis of *B. thai* induced by *P. putida* (Figure 6F).

### T6SS-1 is required for the persistence of *B. thai* in mixed biofilms with *P. putida*

In our plate competition assays, low moisture availability impairs bacterial motility, and artificially enforces close association of *B. thai* with the TDCs. To determine whether T6SS-1 could provide a fitness advantage for *B. thai* under conditions more relevant to its natural habitat, i.e., where nutrients are exchanged and dehydration does not drive interbacterial adhesion, we conducted mixed species flow chamber biofilm assays.

**Figure 7:**
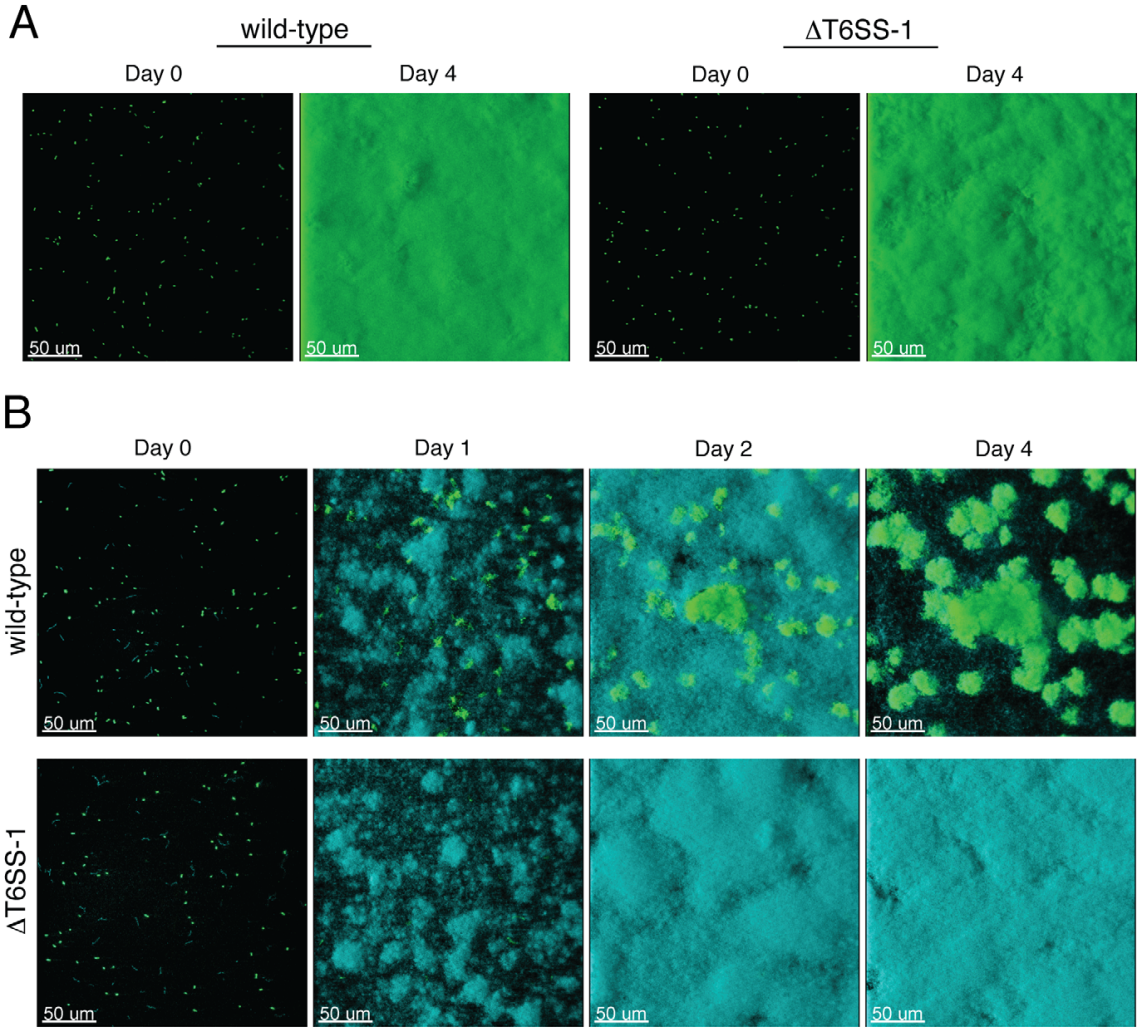
T6SS-1 is required for *B. thai* to persist in mixed biofilms with *P. putida*. Fluorescence confocal microscopy images of *B. thai* (green) and *P. putida* (cyan) biofilm formation in flow chambers. (A) Representative images of monotypic *B. thai* biofilms of the indicated strains immediately following seeding (Day 0) and after four days of maturation. (B) Representative images of mixed biofilms seeded with a 1∶1 mixture of *P. putida* with the indicated *B. thai* strains.

Previous studies in *E. coli* and *V. parahaemolyticus* have implicated T6S in the inherent capacity of these organisms to form biofilms [35], [36]. Furthermore, additional T6SSs are activated during biofilm growth or co-regulated with characterized biofilm factors such as exopolysaccharides [14], [37], [38], [39], [40]. Thus, prior to performing mixed species assays, we first tested whether inactivation of T6SS-1 influenced the formation of monotypic *B. thai* biofilms. Wild-type and ΔT6SS-1 strains adhered equally to the substratum and formed indistinguishable monotypic biofilms that reached confluency after four days (Figure 7A), indicating T6SS-1 does not play a role in the inherent ability of *B. thai* to form biofilms.

Next we seeded biofilm chambers with 1∶1 mixtures of *B. thai* and *P. putida*. In mixed biofilms, the *B. thai* strains again adhered with similar efficiency, however a dramatic difference between the capacity of the strains to persist and proliferate in the presence of *P. putida* became apparent within 24 hours (Figure 7B). At this time point, wild-type *B. thai* microcolonies had expanded and dispersed throughout the *P. putida*-dominated biofilm, whereas *B. thai* Δ*clpV-1* microcolonies had diminished in number. Consistent with the results of our plate assays, *P. putida* growth was not noticeably impacted by the activity of T6SS-1 at early time points in the experiment. As the biofilm matured, wild-type *B. thai* gradually displaced *P. putida*, and by four days after seeding, *B. thai* microcolonies accounted for most of the biofilm volume. These data suggest that T6SS-1 can provide a major fitness advantage for *B. thai* in interspecies biofilms.

## Discussion

Our findings suggest that the highly conserved T6S architecture can serve diverse functions. We found T6SSs within *B. thai* critically involved in two very distinct processes – virulence in a murine infection model and growth in the presence of specific bacteria. The systems involved in these diverse phenotypes, T6SS-5 and T6SS-1, respectively, are distantly related, and cluster phylogenetically with other T6SSs of matching cellular specificity. We were unable to define the function for three of the *B. thai* T6SSs, however their clustering in the H1-T6SS subtree suggests that they could have a role in interbacterial interactions. These systems may not have been active under the assay conditions we utilized, they might be specific for organisms we did not include in our screen, or their activity may not affect proliferation. Phylogenies have proven to be powerful tools for guiding researchers studying complex protein secretion systems [41], [42]. However, determining whether T6S phylogeny holds promise as a general predictor of organismal specificity will require more studies that evaluate the significance of individual systems in both eukaryotic and bacterial cell interactions.

Although *B. thai* is not generally regarded as a pathogen, our data suggest that Burkholderia T6SS-5 plays a role in host interactions that is conserved between this species and its pathogenic relatives, *B. pseudomallei* and *B. mallei* [27], [28], [29], [43]. We postulate that T6SS-5, like many other virulence factors, evolved to target simple eukaryotes in the environment. The benefit T6SS-5 provides the Burkholderia in a mammalian host could have been one factor that allowed *B. mallei* to transition into an obligate pathogen. Based on our results implicating T6SS-1 exclusively in interbacterial interactions, the role of this system in the lifestyle of *B. mallei* is more difficult to envisage. Indeed, the cluster encoding T6SS-1 is the most deteriorated of the T6S clusters of *B. mallei* and is unlikely to function [27]. Of the 13 conserved T6S-associated orthologous genes, 8 of these appear to be deleted in *B. mallei* T6SS-1, however the remaining T6S clusters of the organism are largely intact (0–3 pseudogenes or absent genes).

Of the 33 organisms screened, the effects of *B. thai* T6SS-1 were most pronounced in competitions with *P. putida*, *P. fluorescens*, and *S. proteamaculans*. Whether these organisms are physiologically relevant *B. thai* T6SS-1 targets is not known, however *P. putida* and *P. fluorescens* have been isolated from soil in Thailand [44], [45], and the capacity of these organisms to form biofilms is well documented [46], [47], [48]. *P. putida* and *P. fluorescens* are recognized biological control agents, suggesting that the rhizosphere could be one habitat where antagonism with *B. thai* might occur [49]. Notably, we did not observe T6SS-dependent effects on *B. thai* proliferation in the presence of the five Gram-positive organisms included in our screen. The number and diversity of organisms we tested were too low to ascribe statistical significance to this observation, however it is tempting to speculate that the effects of T6S might be limited to Gram-negative cells. This would not be unexpected given the structural relatedness of T6S apparatus components to the puncturing device of T4 bacteriophage [18], [19], [20].

We found that T6SS-1 allows *B. thai* to proliferate in the presence of the TDCs. This surprising and counterintuitive finding raises the question of what inhibits *B. thai* Δ*clpV-1* growth, and is it an intrinsic (derived from *B. thai*) or extrinsic (derived from the TDC) factor? Our data indicate that the activity or production of this factor manifests in the absence of T6SS-1 function only when a TDC is present and intimate cell contact occurs. If the factor is intrinsic, we postulate that its activity is inappropriately triggered by ΔT6SS-1 in the presence of the TDCs, but that its function serves an adaptive role for wild-type *B. thai*. For example, under circumstances where it is not advantageous for *B. thai* to proliferate, such as when it is exposed to particular organisms, antibiotics, or stresses, this factor could initiate dormancy. There is evidence that T6S components can participate in cell-cell recognition in bacteria. Gibbs *et al.* recently reported the discovery of an “identification of self” (*ids*) gene cluster within *Proteus mirabilis* that contains genes homologous to *hcp* (*idsA*) and *vgrG* (*idsB*) [50]. Inactivation of *idsB* caused a defect in recognition of its parent, resulting in boundary formation between the strains.

If the factor is extrinsic, T6SS-1 might be more appropriately defined as a defensive, rather than an offensive pathway. T6SS-1 could provide defense by either influencing the production of the extrinsic factor within the TDC, such as by repressing expression, or it could provide physical protection against the factor by obstructing or masking its target. If the fitness effect that T6SS-1 provides *B. thai* depends on a specific offensive pathway present in competing organisms, the presence of this pathway in an organism could be the basis for the apparent specificity we observed in our screen. Future studies must address whether the determinants of T6SS-1 effects are intrinsic, extrinsic, or a combination of the two. The design of our competition screen was limited in this regard; we measured T6SS-1 activity indirectly, and we were able to test only a modest number of species. Understanding the mechanism of action of T6SS-1, for example by identifying its substrates, will provide insight into the specificity of the secretion apparatus.

While it is widely accepted that diffusible factors such as antibiotics, bacteriocins, and quorum sensing molecules are common mediators of dynamics between species of bacteria, an analogous cell contact-dependent pathway has yet to be defined [51]. We found that T6S can provide protection for a bacterium against cell contact-induced growth inhibition caused by other species of bacteria. Given that most organisms that possess T6S gene clusters are either opportunistic pathogens with large environmental reservoirs or strictly environmental organisms, we hypothesize that T6SSs are, in fact, widely utilized in interbacterial interactions. Bacteria-targeting T6SSs may be of great general significance to understanding interactions and competition within bacterial communities in the environment and in polymicrobial infections.

## Materials and Methods

### Ethics statement

All research involving live animals was conducted in compliance with the Animal Welfare Act and other federal statutes and regulations relating to animals and experiments involving animals, and adhered to the principles stated in the *Guide for the Care and Use of Laboratory Animals*, National Research Council, 1996. All work involving animals was approved by the Institutional Animal Care and Use Committee at the University of Washington.

### Strains and growth conditions

*B. thai* E264 and *E. coli* cloning strains were routinely cultured in Luria-Bertani (LB) broth or on LB agar at 37°C. All bacterial species used in this study are listed in the legend of Figure 4. The medium was supplemented with trimethoprim (200 µg/ml), ampicillin (100 µg/ml), zeocin (2000 µg/ml), irgasan (25 µg/ml) or gentamicin (15 µg/ml) where necessary. For introducing in-frame deletions, *B. thai* was grown on M9 minimal medium agar plates with 0.4% glucose as a carbon source and 0.1% (w/v) *p*-chlorophenylalanine for counter-selection [52].

### Construction of markerless in-frame deletions of T6SS genes

*B. thai* T6SSs were inactivated utilizing a previously described mutagenesis technique based on the suicide plasmid pJRC115 containing a mutated phenylalanine synthetase (*pheS*) gene for counter-selection [52]. Unmarked in-frame deletions of three to five T6SS genes per T6SS gene cluster (at least two of which are core T6SS genes; see Figure 1) were constructed by splicing by overlap PCR of flanking DNA [53]. The open reading frames were deleted except for 4–8 codons at the 5′ end of the upstream gene and 3′ end of the downstream gene, and the insertional sequence TTCAGCATGCTTGCGGCTCGAGTT was added as previously described [14]. *E. coli* SM10 λ*pir* was used to deliver the deletion constructs into *B. thai* by conjugational mating and transconjugants were selected on LB agar plates supplemented with trimethoprim and irgasan.

### Genetic complementation of Δ*tssK-5* and Δ*clpV-1*

The conserved T6SS genes *tssK-5* (BTH_II0857) and *clpV-1* (BTH_I2958) were deleted using the in-frame deletion mutagenesis technique described above. For single copy complementation, the mini-Tn7 system was utilized [34]. For this, the *B. thai* ribosomal promoter *P*_S12_ sequence was cloned into the suicide vector pUC18T-mini-Tn7T-Tp using complementary oligonucleotides to yield pUC18T-mini-Tn7T-Tp-*P*_S12_ [54]. The *tssK-5* and *clpV-1* open reading frames along with 16–20 bp upstream were amplified and inserted into pUC18T-mini-Tn7T-Tp-*P*_S12_. The resulting plasmids and the Tn7 helper plasmid, pTNS3, were introduced into appropriate deletion strains by electroporation using a previously described protocol [52], [54]. Transposition of the Tn7-constructs into the chromosome of *B. thai* was determined by PCR as described previously [55].

### Construction of fluorescently labeled *B. thai* and *P. putida*

The mini-Tn7 system was utilized to integrate green fluorescent protein (GFP) and cyan fluorescent protein (CFP) expression cassettes into the chromosome of *B. thai* and *P. putida*, respectively [55], [56]. To construct a mini-Tn7 derivative for constitutive expression of GFP, the GFP cassette was amplified from pQBI-T7-GFP (Quantum Biotechnologies) without the T7 promoter region as previously described and inserted into *Kpn*I and *Stu*I sites of pUC18T-mini-Tn7T-Tp-*P*_S12_ [27]. This plasmid was then introduced into relevant *B. thai* strains and insertion of Tn7-GFP into the chromosome was verified as described above. To construct a GFP-labeled Δ*clpV-1* complemented strain, we made use of the fact that two Tn7 insertion sites (*att*Tn7) are present in the genome of *B. thai*. The chromosomally integrated Tn7 Tp^r^ resistance cassette of Δ*clpV-1* complemented was excised using pFLPe2, which expresses a Flp recombinase, before introducing pUC18T-mini-Tn7T-Tp-*P*_S12_-GFP. Insertion of Tn7-GFP into the other *att*Tn7 site was confirmed by PCR as described previously [55], [56]. To engineer CFP labeled *P. putida*, the mini-Tn7(Gm)-CFP plasmid and the helper plasmid pUX-BF13 were introduced into the strain by electroporation as previously described [56].

### *In vitro* growth kinetics

Growth kinetics of *B. thai* strains were measured in LB broth using the automated BioScreen C Microbiology plate reader (Growth Curves) with agitation at 37°C. Three independent measurements were performed in triplicate for each strain.

### Swimming motility assays

Swimming motility of *B. thai* strains was analyzed in 0.25% LB agar. Swimming plates were stab-inoculated with overnight cultures and incubated at 37°C for 48 h. Two independent experiments were performed.

### Murine infection model

Specific-pathogen-free C57BL/6 mice were obtained from Jackson Laboratories (Bar Harbor, ME). MyD88^−/−^ mice were derived by Dr. Shizuo Akira (University of Osaka) and backcrossed for at least 8 generations to C57BL/6 [57]. Mice were housed in laminar flow cages with ad lib access to sterile food and water. The Institutional Animal Care and Use Committee of the University of Washington approved all experimental procedures. For aerosol infection of mice, bacteria were grown in LB broth at 37°C for 18 hours, isolated by centrifugation, washed twice, and suspended in Dulbecco's PBS to the desired concentration. An optical density at 600 nm (OD_600_) of 0.20 yielded approximately 1×10^8^ CFU/ml. Mice were exposed to aerosolized bacteria using a nose-only inhalation system (In-Tox Products, Moriarty, NM) [30]. Aerosols were generated from a MiniHEART hi-flo nebulizer (Westmed, Tucson, AZ) driven at 40 psi. Airflow through the system was maintained for 10 minutes at 24 l/min followed by five minutes purge with air. Immediately following aerosolization, the pulmonary bacterial deposition was determined by quantitative culture of left lung tissue from three to four sentinel mice. Following infection, animals were monitored one to three times daily for illness or death. Ill animals meeting defined clinical endpoints were euthanized. At specific time points after infection, mice were euthanized in order to quantify bacterial burdens and inflammatory responses. To determine bacterial loads, the left pulmonary hilum was tied off and the left lung, median hepatic lobe, and spleen each were removed and homogenized in 1 ml sterile Dulbecco's PBS. Serial dilutions were plated on LB agar and colonies were counted after 2–4 days of incubation at 37°C in humid air under 5% CO_2_.

### Interbacterial growth competition assays

Overnight cultures of *B. thai* and competitor bacteria were adjusted to an OD_600nm_ of 0.1 and mixed 5∶1 (v/v). For competitions using fluorescent strains, 2.5 µl of the mixture was spotted on 3% w/v LB agar and fluorescence was measured after approximately one week following incubation at 30°C. For quantitative competitions using non-fluorescent strains, 10 µl of the mixture was spotted on a filter (0.22 µm; GE Water & Process Technologies) and cells were harvested and enumerated at the indicated time points. Colonies of the competing organisms were distinguished from *B. thai* strains using a combination of colony morphology, growth rate and inherent antibiotic susceptibility.

### Live/dead staining of bacterial cells

Growth competitions of *B. thai* against *P. putida* were performed on filters as described above. At 7.5 h after initiating the experiment, the filters were resuspended in 200 µl LB broth and cell viability was measured using the LIVE/DEAD *Bac*Light Bacterial Viability Kit for microscopy according to the manufacturer's protocol (Invitrogen). The number of dead cells was determined for five random fields per competition using fluorescence microscopy. Two independent experiments were performed in duplicate.

### Flow-chamber biofilm experiments

Biofilms were grown at 25°C in three-channel flow-chambers (channel dimensions of 1×4×40 mm) irrigated with FAB medium supplemented with 0.3 mM glucose. Flow-chamber biofilm systems were assembled and prepared as previously described [58]. The substratum consisted of a 24×50 mm microscope glass cover slip. Overnight cultures of the relevant strains were diluted to a final OD_600nm_ of 0.01 in 0.9% NaCl, and 300 µl of the diluted bacterial cultures, or 1∶1 mixtures, were inoculated by injection into the flow chambers. After inoculation, the flow chambers were allowed to stand inverted without flow for 1 h, after which medium flow was started with flow chambers standing upright. A peristaltic pump (Watson-Marlow 250S) was used to keep the medium flow at a constant velocity of 0.2 mm/s in the flow-chamber channels. Microscopic observation and image acquisition of the biofilms were performed with a Leica TCS-SP5 confocal laser scanning microscope (CLSM) (Leica Microsystems, Germany) equipped with lasers, detectors and filter sets for monitoring GFP and CFP fluorescence. Images were obtained using a 63×/1.4 objective. Image top-down views were generated using the IMARIS software package (Bitplane AG). The flow-chamber experiment reported here was repeated twice, and in each experiment each mono-strain or mixed-strain biofilm was grown in at least two channels, and at least 6 CLSM images were recorded per channel at random positions. Each individual image presented here is therefore representative of at least 24 images.

### T6S phylogenetic tree construction

Annotated genomes were downloaded from the Genome Reviews ftp site (ftp://ftp.ebi.ac.uk/pub/databases/genome_reviews/, January 2010, 926 bacterial genomes (1814 chromosomes and plasmids) [59]. Protein sequences from all genomes were aligned with *rpsblast* [60] against the COG section of the CDD database (January 2010) [61]. Only proteins showing an alignment covering at least 30% of the COG PSSM with an E-value ≤10^−6^ were retained. To avoid any errors in COG assignments, we discarded all hits that overlap with another hit with a better E-value on more than 50% of its length. We considered the following 13 COGs as ‘T6SS core components’: COG0542, COG3157, COG3455, COG3501, COG3515, COG3516, COG3517, COG3518, COG3519, COG3520, COG3521, COG3522, COG3523 [3], [4]. Two genes were considered neighbours if they are separated by less than 5000 bp. Only clusters containing the VipA protein (COG3516) and genes coding for at least five other T6SS core components were included in the analyses. The *Edwardsiella tarda* (EMBL access AY424360) system was added manually because the complete genome sequence and annotation of this organism was unavailable in Genome Reviews.

In three of the 334 T6SS clusters, two VipA coding genes were identified. Manual inspection of two of these clusters in *Acinetobacter baumannii* (ATCC 17978) and *Vibrio cholerae* (ATCC 39541) revealed that they resulted from apparent gene fissions; in both cases we kept the longest fragment corresponding to the C-terminal part of the full length protein. In the third case, *Psychromonas ingrahamii* (strain 37), the two VipA coding genes resulted from an apparent duplication event: one of the two copies showed a high mutation frequency and was discarded. In total, we included 334 VipA orthologs in T6SS clusters. The 334 VipA protein sequences were aligned using *muscle* [62]. Based on this alignment, a neighbour-joining tree with 100 bootstrap replicates was computed using *BioNJ* [63].

## Supporting Information

Figure S1 Dendrogram of T6S phylogeny based on VipA sequences from 334 T6SSs. Color coding is the same as in Figure 1 and is taken from the bacterial taxonomy tree shown in upper left.
(4.21 MB TIF)
