# Supplementary figures and images for: Burkholderia Type VI Secretion Systems Have Distinct Roles in Eukaryotic and Bacterial Cell Interactions
Source: PLoS Pathog. 2010 Aug 26;6(8):e1001068. doi: 10.1371/journal.ppat.1001068 (PMC2928800; doi:10.1371/journal.ppat.1001068)

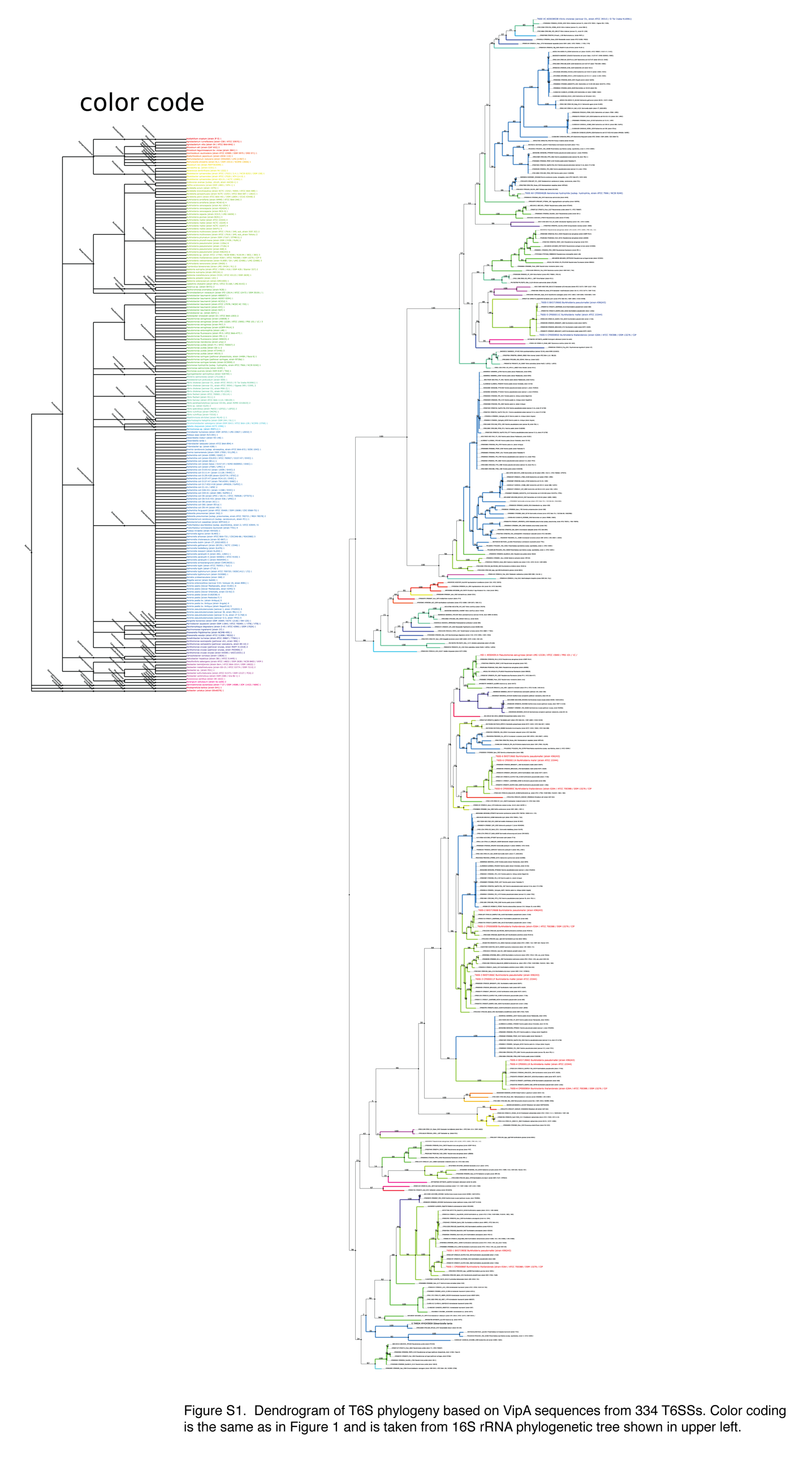

Supplement: Figure S1 — Dendrogram of T6S phylogeny based on VipA sequences from 334 T6SSs. Color coding is the same as in Figure 1 and is taken from the bacterial taxonomy tree shown in upper left. (4.21 MB TIF) [file ppat.1001068.s001.tif]
